# Supplementary material for: Person-Centeredness in Digital Primary Healthcare Services—A Scoping Review
Source: Healthcare (Basel). 2023 May 1;11(9):1296. doi: 10.3390/healthcare11091296 (PMC10178010; doi:10.3390/healthcare11091296)
Supplement: Supplementary file 1 [file healthcare-11-01296-s001.zip › healthcare-2358293-supplementary.pdf]

**File S1. Characteristics of articles included in the scoping review of studies on patient/person-centredness in digital primary health care.**						
**First author Year Country**	**Title**	**Aim/Research question**	**Study design, data collection and analysis**	**Setting and sample, including participants' characteristics**	**Summary of relevant findings**	**Further research need stated**
Ali et al.<br>2021<br>Sweden	Effects of PCC using a digital platform and structured telephone support for people with chronic obstructive pulmonary disease and chronic heart failure: randomized controlled trial	To evaluate the effects of PCC through a combined digital platform and telephone support for people with COPD and CHF	Multicenter randomized trial<br><br>Participants were randomized into either usual care (112/222, 50.5%) or PCC combined with usual care (110/222, 49.5%).<br><br>Intervention: a personal health plan cocreated by the participants and assigned health care professionals.<br>The health care professionals called the participants in the intervention group and encouraged narration to	9 primary care centers<br><br>N=222 patients<br><br>Age: mean 70.8 years (SD 9.4)<br><br>Sex: 103 women and 119 men<br><br>Diagnosis:<br>COPD: 115 (51.8%)<br>CHF: 85 (38.3%)<br>Both COPD and CHF: 22 (9.9%)<br><br>Current smokers: 32	No significant differences were found between the groups at 3 and 6 months<br>The per-protocol analysis of the 3-month follow-up revealed a significant difference in general self-efficacy between the study groups (P=.047), that was not maintained to the 6-months follow-up (P=.24).	To explore which patient at what point in the natural history of the disease would benefit the most and tailor different digital interventions and PCC components to each patient's unique needs.

			establish a partnership using PCC communication skills. A digital platform was used as a communication tool. The usual care group had no follow-up phone conversations. The primary end point, divided into 2 categories (improved and deteriorated or unchanged), was a composite score of change in general self-efficacy and hospitalization or death<br>6 months after randomization.<br><br>Data collection: questionnaires at inclusion, and after 3- and 6-months<br><br>Analysis: statistics	(14.4%)		

Barenfeld et al.<br>2020<br>Sweden	Fro more of an insider: A grounded theory study on patients' experience of a person-centred e-health intervention	To explore the experiences of a person-centred e-health intervention, in patients diagnosed with COPD or CHF	Grounded theory approach<br><br>Intervention: a digital platform and telephone support system for 6 months. The intervention relied on person-centred ethics operationalised through three core PCC components: patient narratives, partnership and shared documentation.<br><br>Data collection: Face to face (n=5) or telephone (n=7) interviews after the intervention.<br><br>Analysis: Charmaz (constructing grounded theory) and NVivo	9 primary care units<br><br>N=12<br>Patients<br><br>Age: 57-81 years (mean 71.4 years)<br><br>Sex: 5 women and 7 men<br><br>Diagnosis:<br>CHF: 1<br>COPD: 9<br>Both CHF and COPD: 2	The core category: «Being welcomed through the side door when lacking the front door keys.»<br>The intervention allowed patients to choose their entry into partnership with health professionals. In contrast, the inclusion of family and friends as partners in the intervention held low priority for patients. The patients experienced the intervention as an effective measure to strengthen self-management processes.<br>Moreover, the patient participants valued their partnership with health care professionals.	None stated

Bekelman et al.<br>2015<br>USA	Primary results of the Patient-Centered Disease Management (PCDM) for heart failure study.<br>A randomized clinical trial	To determine the effectiveness of a collaborative care PCDM intervention to improve the health status of patients with HF	A multisite randomized clinical trial comparing a collaborative care PCDM intervention with usual care in patients with HF.<br><br>Intervention: collaborative care by a multidisciplinary care team consisting of a nurse coordinator, cardiologist, psychiatrist, and primary care physician; home telemonitoring and patient self-management support; and screening and treatment for comorbid depression.<br><br>Data collection:	4 veterans affairs centers<br><br>N=392 Patients<br><br>Age: mean intervention group:67.3 years (SD 9.6), control group: 67.9 years (SD 10.6)<br><br>Sex: male intervention group: n=178 (95.2%), control group:n= 193 (98.0%)<br><br>Diagnosis: HF diagnosis with a heavy symptom burden, impaired functional status and quality of life	There were no significant differences in baseline characteristics between patients; baseline mean KCCQ overall summary scores were 37.9 vs 36.9 (P = .48). There was significant improvement in the KCCQ overall summary scores in both groups after 1 year (mean change, 13.5 points in each group), with no significant difference between groups (P = .97). The intervention was not associated with greater improvement in the KCCQ overall summary scores when the effect over time was estimated using 3-	Fewer deaths were observed in the intervention arm of this trial, and this finding may merit further study in another investigation.<br><br>Rigorous evaluation of disease management programs in HF continues to be necessary.

			The Kansas City Cardiomyopathy Questionnaire (KCCQ) and the Patient Health Questionnaire 9 (PHQ-9) were used at baseline, and at 3, 6 and 12 months after the intervention. In addition, mortality and hospitalization in the same periode were reported.<br><br>Analysis: statistics	Comorbidities:<br>Diabetes mellitus: 50.0%<br>Prior myocardial infarction: 40.6%<br>Chronic obstructive pulmonary disease: 30.2%<br>Obstructive sleep apnea: 43.8%<br>Thirty-eight percent had ejection fraction of less than 40%.	month, 6-month, and 12-month data (P = .74). There were significantly fewer deaths at 1 year in the intervention arm (8 of 187 [4.3%]) than in the usual care arm (19 of 197 [9.6%]) (P = .04). Among those who screened positive for depression, there was a greater improvement in the PHQ-9 scores after 1 year in the intervention arm than in the usual care arm (2.1 points lower, P = .01). There was no significant difference in 1-year hospitalization rates between the intervention arm and the usual care	

					arm (29.4% vs 29.9%, P = .87).	
van der Cingel et al.<br>2021<br>Netherlands	From clinical reasoning to ehealth interventions; a study on how nurses asses care and ehealth in home care	To explore in what way homecare nurses assess eHealth interventions during assessment of care	Explorative, qualitative design<br><br>First, orientation in daily nursing practice was done. Subsequently, the main part of the study took place, using Think Aloud (TA) interview sessions, some of them focussing on the use of classification systems. Finally, a focus group was performed in which preliminary results were discussed.	Homecare<br><br>N=43 Registered nurses<br><br>Age: range 30 to 65 years (average 38)<br><br>Experience in practice ranging between 1 and 17 years (average 6)	Five themes emerged concerning: the holistic view of nurses and importance of putting the client first; dilemmas in care that nurses encounter, specifically when it concerns enhancement of self management; the way clinical reasoning and decision making is done; the perception of eHealth according to homecare nurses; and the opinions they have about eHealth in homecare.	To focus on how nurses can be convinced to assess and use eHealth in a person-centred way and how to discuss this with their clients.
Dhillon et al.<br>2016<br>New Zealand	Designing and evaluating a patient-centred health	To explore how best to design a patient-centric health management system that is	Participants were invited to use Healthcare4Life (made accessible via the Web) at their	Home-health management<br><br>N=43<br>Seniors	Senior healthcare consumers viewed the HMS prototype positively, and experienced a	More health psychology research needs to be incorporated, e.g. by using visual

	management system for seniors	widely available, affordable, extendable by third parties, and is well accepted by seniors	own pace over a six week period. Activities in the system were logged and participants were asked to complete questionnaires at the start of the study, at the end of the third week, and at the end of the sixth week. The questionnaires consisted of the Multidimensional Health Locus of Control (MHLC), the Intrinsic Motivation Inventory (IMI) and the System Usability Scale (SUS).	Age: 60 to 85 years (mean 79, SD = 17.68)	positive change in their attitude towards their health. We identified requirements and challenges for HMSs. In particular, participants indicated that social networking features must have a clear purpose beyond simple broadcasting of emotions and opinions.	information to promote behavioural change. Longer term studies with larger patient populations and more content are necessary to confirm and quantify the long-term health effects of Healthcare4Life.
DiCarlo et al. 2016<br>UK	Patient-centered home care using digital medicine and telemetric data for hypertension: Feasibility and acceptability of	To determine whether daily health decisions, medication use, and/or pharmacologic unresponsiveness	Digital dose forms of valsartan were integrated with passive telemetric data collection and bidirectional communication, and piloted in a	Home care<br><br>N=37 Patients<br><br>Age: 62 years +/- 9 years	Passive detection of DM ingestion was 98% when compared with directly observed dosing. Mean taking and timing adherence	None stated

	objective ambulatory assessment	should be the focus of blood pressure (BP) management	hypertensive population. The objectives included automatic recording, summarization, and communication of: (1) the regularity and pattern of individual medication-taking, daily step count, and daily BP and weight; (2) safety; and (3) patient acceptability.<br><br>Intervention for 6 weeks:<br>Digital Medicine (DM) prototypes consisted of valsartan 80 mg or 160 mg placed in a gelatin hemicapsule with an excipient tablet as a “stopper,” with a poppy seed– sized ingestible sensor (IS) made of	Sex: 14 women and 23 men	rates were 90% and 83%, respectively, and the average step count at a pace of ≥60 steps per minute was 2.0 +/- 1.5 h/ d. An automatic SMS was sent and 100% confirmed for 251 BP and 14 weight values that were not received. Mild and transient WS-related skin irritation was the most common device-related adverse event. There were no serious or unanticipated adverse events. Ninety percent of patients did not mind swallowing a DM capsule, and 75% had a positive	

			foodstuff on its external surface and capable of creating a biogalvanic current on ingestion to alert a wearable sensor (WS) that was worn on the torso. Passive data collection included IS ingestion dates and times, daily step count, BP, and weight. Automatic short message service (SMS) reminders were sent whenever BP or weight values were not received.		overall experience with the system.	
Innominato et al.<br>2018<br>France	Home-Based e-health platform for multidimensional telemonitoring of symptoms, body weight, sleep, and circadian activity: relevance for chronomodulated administration of	To assess the impact of chronomodulated irinotecan fluorouracil-leucovorin and oxaliplatin (chronoIFLO4) delivered at home on the daily life of patients with cancer in real time using a	Patients received chronoIFLO4 fortnightly at home. Patients completed the 19-item MD Anderson Symptom Inventory on an interactive electronic screen, weighed themselves on a dedicated scale, and	Home care<br><br>N=11<br>Patients<br>Age: 48 to 72 years, median 60<br><br>Sex: 6 women and 5 men (45%)	Individual patient compliance ranged from 7% to 100% (median, 55%). Patients expressed overall satisfaction regarding their participation in this pilot experience.<br><br>The most severe MD Anderson	None stated

	Iinotecan, Fluorouracil-Leucovorin, and Oxaliplatin at home—Results from a pilot study	home-based e-Health multifunction and multiuser platform.	continuously wore a wrist accelerometer for CircAct and sleep monitoring. Daily data were teletransmitted to a specific server accessible by the hospital team. The clinically relevant CircAct parameter dichotomy index I < O and sleep efficiency (SE) were calculated. The dynamic patterns over time of patient-reported outcome measures, BWC, I < O, and SE informed the oncology team on tolerance in real time.<br><br>Patients were interviewed informally by the nursing staff at the outset of the study period.	Diagnosis: Advanced or metastatic colorectal (n = 5) or pancreatic (n = 6) cancer	Symptom Inventory scores were: interference with work (mean: 5.1 of 10) or general activity (4.9), fatigue (4.9), distress (4.2), and appetite loss (3.6). Mean BWC was −0.9%, and mean SE remained > 82%. CircAct disruption (I < O ≤ 97.5%) was observed in four (15%) cycles before chronoIFLO4 start and in five (19%) cycles at day 14	

Johansdottir et al.<br>2021<br>Iceland	Rural patients' experience of education, surveillance, and self-care support after heart disease related hospitalisation: a qualitative study	To explore the experiences of people with coronary heart disease, living in rural Iceland regarding patient education, surveillance, and self-care support.	Qualitative design<br><br>Semi-structures individual interviews 6 to 12 months after hospital discharge following a cardiac event.<br><br>Analysis: systematic text-condensation	Home care<br><br>N=14<br>Patients<br><br>Age: mean 67 years (SD 9.0). Range 52-79 years.<br><br>Sex: 6 women and 8 men<br><br>Diagnosis:<br>Acute PCI: 10<br>Elective PCI: 4	Three main themes;<br>- «Education and support» describes inadequate patient education and support from health-care professionals after discharge from hospital, and how the internet was the main information source supplemented with spouse's and family support.<br>- «Local healthcare services» describe the lack of and importance of access to healthcare professionals, stable services, and underutilisation of telemedicine and primary healthcare in the local area.<br>- «Self-care behaviour» describes the lack	Further research is needed on self-care of cardiac patients living in the Arctic, and how it is affected by their special environmental situation. Also, more intervention studies are needed to explore how e-health and telemedicine can possibly be used to improve patient education and support self-care in the Arctic

					of professional support with lifestyle changes and how the participants manage self-care as well as their attitudes towards the disease.	
Kim et al.<br>2020<br>US	Enabling self-management of a chronic condition through patient-centered coaching: A case of an mHealth diabetes prevention program for older adults	Research question: What patterns of PCC strategies occur in an mHealth-based diabetes prevention programs (DPP) for older adults?	Analysis of user-coach communication in Noom Coach for DPP (Noom) a commercial mHealth application that offers lifestyle intervention program for preventing diabetes in older adults (aged 65 or older) during the 16-week period of the program<br><br>Engineers extracted the anonymized textual data of user-coach conversation. The communication data from the in-app chat system were	Home-prevention<br><br>N=30<br>Older adults<br><br>Age: mean 68.17 (SD 2.63) years<br>Range: 65-74 years<br><br>Sex: 20 women and 10 men.<br><br>N=33<br>Coaches	Four PCC strategies were employed by coaches: (a) triggering reflections on users' routinized habits, (b) jointly determining a measurable health goal, (c) facilitating self-evaluations on recent behavior change, and (d) tailoring programs to adapt to users' lifestyle and health status. To advance these strategies, coaches utilized various	Further examinations of the differences in coaching practices may allow us to generate an in-depth understanding of message design strategies, efficacy of coaching, and concomitant intervention outcomes.

			converted to Word documents, generating single-spaced texts of 549 pages that include textual conversations between 30 users and 33 coaches. In a few user cases, users were assigned to a new coach during the program		mHealth features that helped them (a) engage in data-driven coaching, (b) increase situational awareness of users' health conditions and routines, (c) provide continuous support to users through regular and spontaneous in-app chats, and (d) foster user autonomy and engagement.	
Klein et al. 2017<br>US	The Veteran-initiated electronic care coordination: a multisite initiative to promote and evaluate consumer-mediated health information exchange	The goals were twofold: 1) train dual-use rural veterans to use Veteran Affairs' (VA) My HealtheVet Blu Button capabilities to promote consumer-mediated health information exchange (HIE) of their VA Countinuity of Care	Pilot study.<br><br>Implementation process was multifaceted involving: 1. Engagement of VA facilities and rural community healthcare organizations to develop optimal processes for information	Veteran affairs<br><br>N=620 Veterans<br><br>Age: mean age 67 years (SD=10.6)<br><br>Sex: 584 men (94%)<br><br>N=277 community	After training, 78% reported the CCD would help them be more involved in their healthcare and 86% planned to share it regularly with non-VA providers. Veterans (n = 256) then attended 277 community appointments. Provider responses	None stated

		Document (CCD) with their non-VA care providers, and 2) to evaluate if the availability of VA information at a community clinical encounter impacted care recieved.	exchange, 2. Veteran engagement and training in health information sharing, and 3. methods for evaulating patient and provider impact of this sharing.<br><br>Veterans trained by mail, n=496<br>Veterans trained in-person=100<br>Training method not known, n=24<br><br>Patients and non-VA providers completed surveys on their experience.	provider visits were evaluated	from these appointments (n = 133) indicated they were confident in the accuracy of the information (97%) and wanted to continue to receive the CCD (96%). Ninety percent of providers reported the CCD improved their ability to have an accurate medication list and helped them make medication treatment decisions. Fifty percent reported they did not order a laboratory test or another procedure because of information available in the CCD.	

Radhakrishnan et al.<br>2016<br>US	Unsustainable home telehealth: a Texas qualitative study	To employ a sociotechnical systemic approach to explore the reasons for the initial adoption and eventual decline of the decade-long Texas home health agency (TxHHA) telehealth program and to identify barriers to and facilitators for sustaining home telehealth program<br><br>Analysis: conventional content analysis	Semi-structured individual interviews	Home care<br><br>N=13<br>Home health staff<br><br>Age:<br>31–40 years (8%)<br>41–50 years (23%)<br>>50 (69%)<br><br>Sex: 13 women<br><br>N=9<br>Patients<br><br>Age:<br>50–59 years (22%)<br>60–79 years (33%)<br>>79 years (44%)<br><br>Sex: 7 women (78%) and 2 men (22%)	Five themes representing the decline of the Texas home telehealth program: 1)impact on patient-centered outcomes; 2) impact on cost-effectiveness; 3) patient–clinician and interprofessional communication; 4) technology usability; 5) home health management culture.<br>Lack of significant impact on patient outcomes, in addition to financial, technical, management, and communication-related challenges, adversely affected the sustainability of this home telehealth program.	Future research should explore data visualization techniques to enable efficient communication of telehealth data, patients' contextual information, and their correlations with health status among clinicians. Research is also needed on patients', nurses', and physicians' decision-making processes and abilities in disease management when informed by telehealth data. Also, an interdisciplinary collaborative planning approach that involves end users of home health nurses and patients as well as physicians can inform design of

						future telehealth interventions that is effective, sustainable, and informed by users need and current reality.
Smaradottir et al.<br>2020<br>Norway	How to enhance digital support for cross-organisational health care teams? A user-based explorative study	To validate and verify the prerequisites for health<br>care systems run with patient-centred service models<br>Research questions:<br>1) how can a digital approach support the communication processes and information needs in cross-organisational health care teams?<br>2) what technical infrastructure can be recommended for a digital approach supporting the information flow in	Explorative study<br><br>Qualitative interviews and observations<br><br>First phase of the data collection:<br>(1) a focus group<br>(2) a semistructured interview in pair, and (3) an individual semistructured interview<br><br>Second phase:<br>A field study with observations of the communication procedures and the technology use in the staff room. Two main technical	Health organisation<br><br>Phase 1:<br>N= 5 in one focusgroup:<br>4 nurses and 1 department manager<br><br>A nurse-physiotherapist pair<br><br>N=1 physician Individually<br><br>Phase 2:<br>N=4 nurses individually<br><br>N=4 nurses in a focus group	Three categories identified: (1) cross-organisational information and workflow, (2) constraints in digital support, (3) proposed digital approach for cross-organisational teamwork.<br><br>The evaluation showed a lacking interoperability between the digital systems and a limited support for cross-organisational teamwork, causing raised manual efforts to maintain	It is suggested to address further research on other patient-centred cross-organisational settings using interoperable information cloud and focusing on the hosting and governing of it, and the information sharing across different platforms

		cross-organisational health care teams? 3) what are the benefits and constraints of the digital approach for the information flow in cross-organisational health care teams?	systems were used, and a thorough demonstration was made of both. Five interviews were made: (1) four semistructured individual (2) a focus group.<br><br>Third phase:<br>a field study was made in the staff room, observing communication and digital support. Five interviews were made: (1) four semistructured individual<br>(2) a focus group<br><br>To complete the data collection, an elderly patient was interviewed at home focusing on communication procedures with the team.	Phase 3:<br>N=4 staff (not specified)<br><br>N=1 patient	the information flow. Tools for coordination and planning across organisations were lacking. To enhance the situation, principles for a cloud-based health portal are proposed with a shared workspace, teamwork functionality for cross-organisational health care teams, and automatic back-end synchronisation of stored information.	

BP=Blood pressure. CCD= Countinuity of Care Document. CHF=Chronic heart failure. COPD=Chronic obstructive pulmonary disease. HIE= Health information exchange. PCC=Person-centred care. PCDM= Patient-Centered Disease Management. VA=Veteran affairs.
